# Impact of Biosecurity on Production Performance and Antimicrobial Usage in Broiler Farms in Cameroon

**DOI:** 10.3390/ani15121771

**Published:** 2025-06-16

**Authors:** Stephane D. Ziebe, Ronald Vougat Ngom, Adonis M. M. Akoussa, Henry P. Bogning, Henriette A. Zangue

**Affiliations:** 1 Department of Animal Production, School of Veterinary Medicine and Sciences, University of Ngaoundere, Ngaoundere P.O. Box 454, Cameroon; ziebestephane@gmail.com (S.D.Z.); adonismedardmartial@gmail.com (A.M.M.A.); 2 SPC (Société des Provenderies du Cameroun), Yaoundé P.O. Box. 17035, Cameroon; peguybogning@gmail.com; 3 National School of Agro-Industrial Sciences Ngaoundere, Ngaoundere P.O. Box 454, Cameroon; hzangue42@gmail.com

**Keywords:** antibiotic, antimicrobial resistance, disease prevention, chicken, FCR, poultry, Africa

## Abstract

The broiler industry is the most developed livestock sector in Cameroon. This study aimed to evaluate the relationship between biosecurity implementation with production performance and antibiotic usage in broiler farms in Cameroon. Data concerning biosecurity, production performance (average daily gain or ADG, mortality rate, feed conversion ratio or FCR, and performance index or PI), and antimicrobial usage (AMU) were collected in 57 farms in the Adamawa and North regions. The average total biosecurity score of broiler farms was 52/100. ADG (46.54 ± 5.18 g versus 43.80 ± 4.16 g), FCR (1.59 ± 0.61 versus 1.75 ± 0.58), mortality rate (2.47% versus 6.65%), and PI (339.21 ± 105.79 versus 268.22 ± 101.09) were statistically better in farms with good biosecurity. The majority of antibiotics used (55.2%) were classified as critically important for human medicine, with 83.9% of antibiotics underdosed/overdosed. No correlation was found between biosecurity and AMU, although there was a trend towards reduced use in farms with good biosecurity. The misuse of antibiotics will result in an increased development of antimicrobial resistance, which can be transmitted to humans. This study highlights the importance of biosecurity in improving poultry performance and reducing AMU. Continuous training and awareness-raising efforts among farmers on the importance of biosecurity are needed to reduce AMU and improve farmers’ profitability.

## Simple Summary

In African countries, poultry plays an important role in terms of human nutrition and the economy. Due to the lack of strict legislation, antimicrobials are extensively used to treat diseases, the main hindrance to the sector. Among the solutions to minimize the risk of disease, strengthening biosecurity is the key strategy. This study aimed to evaluate the relationship between biosecurity implementation and production performance, as well as antibiotic usage in broiler farms in Cameroon. Data concerning biosecurity, production performance, and antimicrobial usage (AMU) were collected in 57 farms between April and October 2024. The average total biosecurity score of broiler farms was moderate. The broiler performance was better in farms with good biosecurity. The majority of antibiotics were misused during treatment. No association was found between biosecurity and the amount of antibiotics used, although there was a trend towards reduced use in farms with good biosecurity. The misuse of antibiotics will result in an increased development of antimicrobial resistance, which can be transmitted to humans. This study highlights the importance of biosecurity in improving poultry performance and reducing AMU. Continuous training and awareness-raising efforts among farmers on the importance of biosecurity are needed to reduce AMU and improve farmers’ profitability.

## 1. Introduction

In Cameroon, the second highest poultry producer in West and Central Africa [1], the poultry sector accounts for 4% of the Gross Domestic Product and plays an important role in food security and providing employment for both urban and rural populations [2]. However, infectious diseases such as highly pathogenic avian influenza, Newcastle disease, and infectious bursal disease represent one of the major challenges in the sector [1,3]. These diseases can significantly reduce the production performance of chickens, resulting in serious economic loss. Decreased body weight gain and increased feed conversion ratio (FCR) due to poultry coccidiosis have been reported by Rahmani et al. [4] in Algeria. Kouam et al. [5] reported 24,668 poultry deaths after the 2016 outbreaks of highly pathogenic avian influenza virus in Cameroon. Colibacillosis has been associated with high mortality, increased FCR, and decreased meat production in many studies [6,7]. Rahmani et al. [4] predict economic losses of approximately £86.7 million due to coccidiosis in chickens in Algeria, with 75% of the cost attributed to morbidity.

Due to this serious impact, many poultry farmers in low- and middle-income countries (LMIC) extensively use antimicrobials to treat or prevent poultry diseases [8,9]. Unfortunately, farmers who assume the responsibility of treating their animals have insufficient knowledge regarding antimicrobial usage (AMU), thus misusing them [9,10]. The increased use and misuse of antimicrobials by chicken farmers are attributed to the lack of regulatory policies in the sector, the lack of veterinarians, the widespread accessibility and availability of these drugs, which are sometimes illegally sourced [11,12,13]. In addition to this, sometimes the drugs administered are of substandard quality [14,15]. All these factors, together, increase the chance of failure of antibiotic therapy, and the emergence and spread of antimicrobial resistance (AMR) in both livestock and humans [16,17], contributing to the higher burden of AMR recorded in LMIC [18]. AMR is one of the most important global health challenges in the 21st century that could be associated with an estimated 8.22 [19] to 10 million deaths [20] globally by 2050.

To reduce the extensive misuse of antimicrobials in poultry farms and control AMR in LMIC, preventive measures such as biosecurity must be prioritized. Biosecurity refers to a set of measures aimed at reducing the risk of introduction, establishment, and spread of diseases, infections, or infestations from or within an animal population [21,22]. In addition to controlling diseases [23], biosecurity has been associated with increased production [24,25] and reduced AMU [26] in broiler farms in high-income countries. In Cameroon, previous works have separately assessed biosecurity, technical performances, and AMU in broiler farms [9,27,28] but not the association of biosecurity with production performance and AMU. This study aimed to assess the impact of biosecurity on production performances and AMU in broiler farms in Cameroon.

## 2. Materials and Methods

### 2.1. Study Area and Design

**Figure 1:**
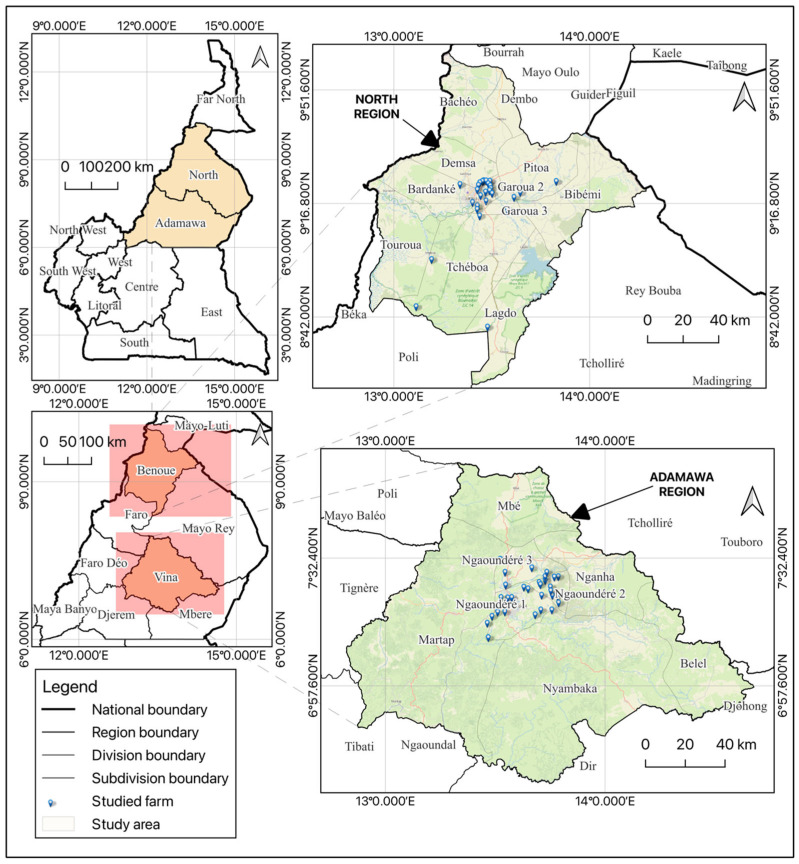
Distribution of broiler farms surveyed in the Adamawa and North regions (Cameroon).

This longitudinal study was carried out from April to October 2024 in broiler farms located in the Adamawa (6th and 8th degrees of North latitude and 11th and 15th degrees of East longitude) and North (8th and 10th degrees of North latitude and 12th and 16th degrees of East longitude) regions of Cameroon. Both regions are from two out of the five agro-ecological zones, named the Sudano-sahelian zone and the Guinea Savanna for North and Adamawa, respectively. Adamawa is covered by a humid tropical highland climate. The average annual rainfall in this area is between 1400 and 1755 mm. The North Region has a tropical climate, with annual rainfall above 900 mm and a 6-month dry season. The transition from Adamawa in the south is abrupt, so average temperatures appear to be all the higher. Adamawa and North are among the poorest regions of the country. For this reason and the rapidly increasing population due to the creation of many private and public higher Schools/Universities, many people are getting involved in broiler production. Broiler production is becoming an important activity in these regions because of the short breeding period (45 days) with less labor intensity, fast growth, low feed conversion ratio, and small land requirements. In addition, poultry meat represents an important source of cheap protein. Furthermore, the Ministry in charge of livestock has encouraged chicken production in these regions by implementing many projects related to this sub-sector. The majority of farms in these two regions are small to medium-scale commercial production systems. Figure 1 presents the distribution of broiler farms visited in this study. Authorizations to carry out this study were obtained from the regional delegations in charge of livestock (N^0^ 0047/24/L/RN/DREPIA/SRSV) and the scientific research and ethics committee of the School of Veterinary Medicine and Sciences of the University of Ngaoundere (2024/061UN/ESMV/DAARCS/SSFC of 27 March 2024).

### 2.2. Data Collection

Due to the lack of a list of poultry farmers, the livestock services in both study regions provided us with the lists of chicken feed companies operating locally. Four companies were randomly selected from the lists. The selected companies were then contacted, and the study objective was explained to the manager. Upon obtaining the approval of the companies, broiler farmers who purchased both chicks and feed from these suppliers were recruited. It is noteworthy that farmers in the study regions generally do not have any formal contracts with chicken feed suppliers and often. Hence, decide where to purchase their day-old chick and feed when the need arises. This is due to the absence of breeder farms in the study area.

For farmers to be included in this study, they needed to have at least 200 broilers on their farm and with a minimum of two years’ experience in raising broilers. Recruitment was performed progressively as farmers arrived at the companies to buy chicks or feed. Once a farmer agreed to participate in the study, a follow-up appointment was scheduled. In total, 57 farms were included in the study. This sampling strategy and sample size minimized variability and improved the feasibility of follow-up.

Data collection performed by a well-trained final-year veterinary student during visits to each farm was conducted under the supervision of an expert researcher. Each farm was visited four times during the study. The first time, without providing a lot of details, the objective of the study and the working methodology were explained to the farmer. Participants were also assured of the confidentiality of the data they would provide and the anonymity of their individual identities. The remaining three visits were in line with the key phases of the production cycle and took place on days 15, 30, and 45. The cycle was divided as follows: the starter phase from 1 to 15 days, the growth phase between 16 and 30 days, and the finishing phase from 31 to 45 days [29].

#### 2.2.1. Biosecurity Assessment

The biosecurity level of each farm was assessed during the second visit to the farm using the broiler Biocheck.UGent^TM^ questionnaire (https://biocheckgent.com/en/questionnaires/broilers, accessed on 5 January 2024) as described by Gelaude et al. [24]. This questionnaire contained question related to external (purchase of 1 d-old chicks, off-farm movements of live animals, feed and water supply, removal of manure and dead birds, entrance of visitors and personnel, supply of materials, infrastructure and biological vectors, location of the farm) and internal (disease management, cleaning and disinfection, materials, and measures between compartments) biosecurity measures as deeply described [24]. All the questions asked for each subcategory of biosecurity measures can be found in the questionnaire (https://biocheckgent.com/sites/default/files/2020-02/Broilers_EN.pdf, accessed on 5 January 2024). The questionnaire was filled out during an interview with the farm manager or owners, followed by direct observation on the farm. Once the questionnaire was completed, the information was entered into the online Biocheck.UGent^TM^ database (https://biocheckgent.com/en, accessed on 10 April 2024) to obtain scores for the various subcategories (purchase of 1 d old chicks, feed and water supply, cleaning and disinfection, etc.) of biosecurity measures. This risk-based biosecurity quantification system generates scores associated with the levels of external and internal biosecurity. Scores range from 0 (=total absence of any biosecurity measure) to 100 (=full application of all available biosecurity measures). The overall biosecurity level of a farm is measured by a weighted average of the external and internal biosecurity scores. Detailed information on Biocheck.UGent™ system for the broilers is provided by Gelaude et al. [24].

#### 2.2.2. Evaluation of Antibiotic Usage

Data on antibiotic use were collected through a monitoring sheet designed for this purpose. This sheet, available from the author on request, allowed for the collection of data on the different antibiotics used (name, class, active substance, quantity administered, quantity of water used, route of administration, duration of the administration, etc.), indications (curative, preventive) and reasons for usage. During the first visit to the farm, the farmers were trained to fill out the form. Usually, farmers visit their farms at least two times per day (morning and afternoon), and they have workers who spend the whole day on the farm. Both the farm owner and the workers who spent the whole day on the farm (generally in charge of the administration of treatment) were trained. When that was not possible, only the workers received the training to collect the data. The form filled by the farmers was checked at each visit to the farm.

The metrics calculated to assess antibiotic use include treatment incidence (TI), used daily dose (UDD), Animal daily dose (ADD), and the amount used to produce one kilogram of meat.

- The UDD describes the amount of active substance actually administered to the animals in mg/kg. It was calculated by dividing the amount of antimicrobial compound administered (mg) by the number of broilers multiplied by the average weight at the time of treatment (treatment duration) [30];
- The ADD, which is the assumed average dose per day per kilogram of chicken of a specific drug, was collected from the drug’s instruction leaflet;
- The amount used to produce one kilogram of meat was obtained by dividing the total amount of active substance (AS) used by the total weight of the subjects during treatment;
- The UDD/ADD ratios were calculated to assess the correctness of dosage; 0.8 ≤ UDD/ADD ≤ 1.2 was considered as correct dosage, and 0.8 < UDD/ADD and UDD/ADD > 1.2 were considered as underdose and overdose, respectively [30,31];
- Treatment incidence for chickens is defined as the number of chickens per 1000 that were treated daily with a UDD or ADD [32]. TI was calculated using the following formula:

$$TI=\frac{Total\;amount\;of\;antimicrobial\;administered\left(mg\right)}{ADD\;ou\;UDD\left(\frac{mg}{Kg}\right)\times Number\;of\;days\;at\;risk\times Weight\;of\;chickens\;on\;farm\;\left(kg\right)}\times 1000$$

where

- The number of days at risk corresponds to the duration in days during which the broiler may have been exposed to one or more treatments.
- The weight of the subjects was obtained by multiplying the number of chickens by their average weight.

#### 2.2.3. Evaluation of Production Performance of Broiler Farms

Data on production performances were collected during the visits to the farm through a monitoring sheet designed for this purpose. This sheet in French, available from the author on request, allowed for the collection of information on the place of purchase of feed, the quantity purchased and the quantity used, the weight of the animal, the number of dead animals, and the number of animals sold according to the different phases of production. To obtain the weight of animals, in each production phase, 5% of broilers were randomly selected and weighed using a digital scale (model AB-J99). Average daily gain (ADG), mortality rate, FCR, and performance index (PI) were calculated from the recorded data as described below.

- Mortality rate

It was calculated according to the formula suggested by Gelaude et al. [24].

$$Mortality\;rate=\frac{M1+S1}{n1}\times 100\%$$

*M*1 = number of animals dead at time T1;

*S*1 = number of animals euthanized at time T1; *n*1 = number of subjects at time T1.

- Feed Conversion Ratio

It was calculated using the following formula:

$$FCR=\frac{Amount\;of\;feed\;consumed\;during\;period\;t}{Weight\;gain\;during\;period\;t}$$

- Average daily gain

It was calculated according to the formula below [33].

$$ADG=\frac{Final\;weight-Initial\;weight}{Duration\;of\;the\;phase}$$

- Performance Index

It was calculated with the following formula [24]:

$$PI=\frac{(100-total\;Mortality)\times ADG}{10\times FCR}$$

### 2.3. Statistical Analysis

The information obtained on biosecurity and the quantity of antibiotics was entered into a Microsoft Excel (2013) spreadsheet and then analyzed with the Statistical Package for Social Sciences (SPSS) 20 software. A total biosecurity score of less than 50 was considered low, while farms with a total biosecurity score between 50 and 100 were assigned good biosecurity. To evaluate the impact of AMU in farms on public health, the antimicrobial families were categorized according to recent WHO classification into four main groups based on their medical importance and the risk of AMR: highest priority critically important antimicrobials (HPCIA), critically important antimicrobials (CIA), highly important antimicrobials (HIA), and important antimicrobials (IA) [34]. Descriptive statistics were used to highlight farm characteristics, farmer profiles, and AMU. One-way analysis of variance followed by Duncan’s comparison test was used to compare more than two means. Student’s test was used to compare two means, while Pearson’s test was used to examine the association between two variables. The correlation was considered weak when R^2^ < 0.3, moderate when 0.3 ≤ R^2^ < 0.5, strong when 0.5 ≤ R^2^ < 0.7 and very strong when R^2^ ≥ 0.7. For all statistical analyses, the significance level was set at 5%.

## 3. Results

### 3.1. Profiles of Respondents and Characteristics of Farms

Table 1 summarizes the profile of the farmers who participated in this study. Among the 57 farmers surveyed, the majority were men (78.9%) and were between 31 and 40 years old (60.1%). Most of the respondents had no training in poultry farming (54.6%) and did not have poultry farming as their main activity (75.9%). In addition, 82.6% of farmers had less than 10 years of experience in poultry farming. The number of broilers reared by farmers varied from 200 to 2,500 per cycle, with most farms (84.3%) housing between 200 and 500 birds. Less than 10% of farms had more than 1,000 broilers. The majority of farmers have at a maximum of two barns (82.3%).

### 3.2. Biosecurity in Broiler Farms

The percentage of farms with a good level of biosecurity was high (68.4%). Table 2 shows that the average biosecurity score of broiler farms in the study area was 52 out of 100 (range 28–71). Average internal and external biosecurity scores were 56 (range 31–73) and 50 (range 23–72), respectively. The biosecurity measures related to feed and water had the lowest score (30, range 19–77), while biosecurity measures related to materials and measures between compartments were the most adopted in farms (78, range 0–100). The overall biosecurity score was significantly higher in the Adamawa (55.1 ± 10.1) than in the North Region (49.0 ± 10.7) (*p* = 0.039). This trend also concerned both internal biosecurity (58.6 ± 8.1 for Adamawa and 53.5 ± 9.3 for the North, *p* = 0.034).

### 3.3. Antibiotic Usage

#### 3.3.1. Quantity of Antibiotics Used

**Figure 2:**
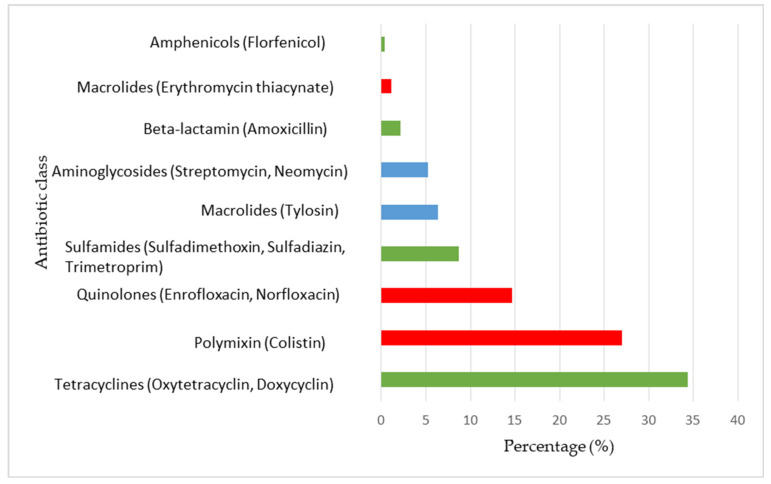
Medical importance and distribution of the antibiotics used in broiler farms in Adamawa and North regions (Cameroon). Red: highest priority critically important antimicrobials; Blue: critically important antimicrobials; Green: highly important antimicrobials.

All the farms included in this study used antibiotics during broiler production. In total, 23 commercial antibiotics containing 15 different AS were used during the study period in broiler farms in Adamawa and North Regions in Cameroon. The majority of drugs contained one (65.2%) or two (34.8%) AS. In the 57 farms, a total of 230 AS were used and administered through drinking water. Among the total AS recorded, Oxytetracycline (29.6%, 95% CI: 23.7–35.5) was the most commonly used followed by colistin (27.0%, 95% CI: 21.3–32.7), enrofloxacin (10.0%, 95% CI: 6.1–13.9) and tylosin (6.5%, 95% CI: 3.3–9.7). In addition, 44.8% (95% CI: 38.4–51.2) of the AS were categorized as HIA while 55.2% (95% CI: 48.8–61.6) were classified as critically important for human medicine, with 43.0% (95% CI: 36.6–49.4) of HPCIA and 12.2% (95% CI: 7.9–16.4) of CIA (Figure 2). In terms of the class of antibiotics, Tetracyclines were the most used, representing 34.3% of the total AMU. Polymyxins, represented by colistin (27.0%) and quinolones (14.7%), were the second and the third most used classes, respectively.

**Figure 3:**
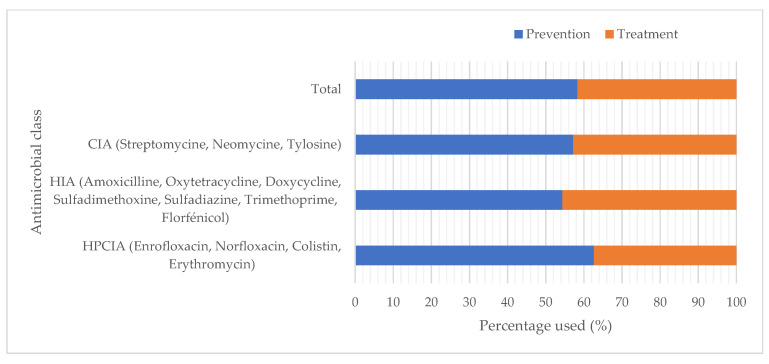
Distribution of the classes of antibiotics (according to WHO classification) used in broiler farms according to the indication. HPCIA: highest priority critically important antimicrobials; CIA: critically important antimicrobials; HIA: highly important antimicrobials.

The majority of antibiotics (58.3%) was used for disease prevention. The rest was mainly used for treatment of digestive (21.2%) and respiratory (20.5%) diseases. Nearly two-thirds of HPCIAs (62.6%) and more than half of CIAs (57.1%) were used to prevent diseases (Figure 3).

Table 3 presents the TI of antibiotics in the study area. It shows that the average values of 85.5 daily doses per 1000 broilers for TI_UDD_ (range: 22.2–111.1) and 47.3 daily doses per 1000 broilers for T_IADD_ (range: 0.004–920.9) were recorded with a high significant difference among TI_UDD_ and T_IADD_ (*p* < 0.001) was observed between TI_UDD_ and T_IADD_. Streptomycin had the highest TI_UDD_ (100) while Florfenicol recorded the lowest TI_UDD_ (44.44). Sulfadimethoxine had the highest T_IADD_ (272.77) while erythromycin recorded the lowest T_IADD_ (3.51).

Table 4 shows the amount of antibiotics (mg) used per kg of broiler meat produced. In general, the amount of antibiotic used per kg of broiler produced was 784.4 mg/kg. To produce a kilogram of broiler meat, Doxycycline was the most AS used (198.0 mg), followed by amoxicillin (105.0 mg). The molecules least used to produce a kilogram of meat were streptomycin (2.0 mg) and erythromycin thiacynate (6.8 mg).

Table 5 presents the correctness of dosage during antibiotic treatment administered by farmers. Overall, 83.9% of AS were misused, with 77.8% of underdosing treatment. None of the antibiotics were always correctly used by broiler farmers.

#### 3.3.2. Association Between Biosecurity and Antibiotic Usage

The results in Table 6 show that the TI_UDD_ (68.41 ± 38.73) and the quantity of antibiotic used to produce one kg of meat (35.66 ± 15.10 mg) were lower in farms with good biosecurity compared to farms with poor biosecurity (75.23 ± 40.13 and 47.75 ± 26.20 mg, respectively), but this difference was not statistically significant (*p* > 0.05). No correlation was found between AMU and the level of biosecurity on the farm (Table 7).

### 3.4. Production Performances

#### 3.4.1. Performance of Broiler Farms

Among the farms included in this study, 56.14% (32/57) used feed from the selected companies during at least two of the three production phases. 54 out of 57 farms (94.7%) used the Ross breed while the rest used Hubbard. Table 8 presents the production performances of the farms surveyed. The overall ADG and FCR were 45.71 ± 5.14 g and 1.59 ± 0.48, respectively. Both FCR and ADG were best during the grower and finisher phases. The total mortality rate per farm in the study area was 3.55 ± 3.30%, with a significantly high mortality recorded during the starter phase (1.77 ± 1.28%). The average performance index was 317.76 ± 115.56.

#### 3.4.2. Relation Between Biosecurity and Production Performances

In general, broiler farms with a good biosecurity level (≥50) showed better production performance as detailed in Table 9. The total ADG was significantly (*p* = 0.034) higher in farms with good biosecurity (46.54 ± 5.18 g) compared to those with poor biosecurity (43.80 ± 4.16 g). Similarly, significantly low FCR (1.50 ± 0.35 vs. 1.72 ± 0.57; *p* = 0.026) and Mortality rate (2.47% vs. 6.65%; *p* < 0.001) were recorded in farms with good biosecurity levels. Likewise, PI in farms with good biosecurity (339.21 ± 105.79) was statistically (*p* = 0.015) higher compared to that in farms with poor biosecurity (268.22 ± 101.09). The odds ratio (OR) between biosecurity and mortality was 1.19 (1.13–1.26).

**Figure 4:**
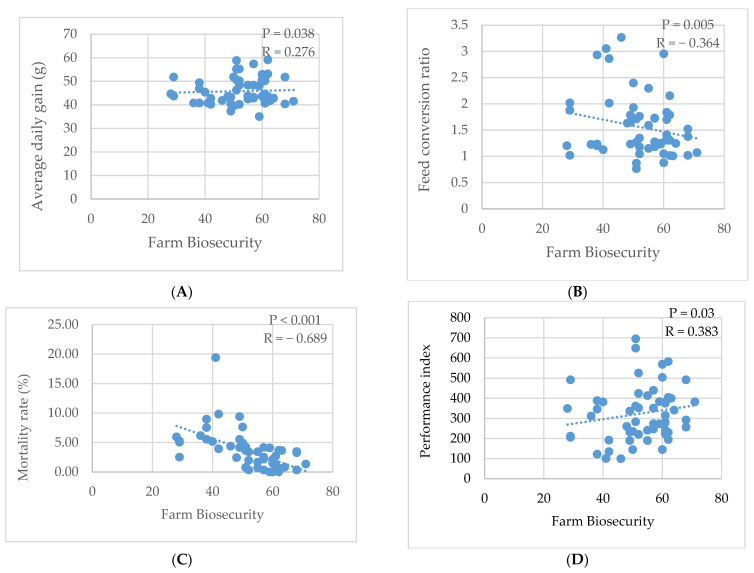
Correlation between biosecurity and production performance in broiler farms. (**A**): Correlation between biosecurity and average daily gain; (**B**): Correlation between biosecurity and feed conversion ratio; (**C**): Correlation between biosecurity and mortality rate; (**D**): Correlation between biosecurity and performance index.

As can be seen in Figure 4, a strong correlation (R^2^ = −0.689, *p* < 0.001) was recorded between the level of biosecurity and the mortality rate in broiler farms. Results showed that there was a low positive correlation (R^2^ = 0.276, *p* = 0.038) between ADG and biosecurity (Figure 4A). On the other hand, FCR decreased moderately (R^2^ = −0.364, *p* = 0.005) when biosecurity increased (Figure 4B). A moderate positive correlation (R^2^ = 0.383, *p* = 0.030) between the performance index and biosecurity was noted (Figure 4C,D).

## 4. Discussion

This study was conducted with the aim of assessing the relationship between biosecurity and production performance as well as antibiotic use in broiler farms in Cameroon. Biosecurity is one of the essential components of the One Health concept [35]. It is considered essential in this context, as it aims to prevent diseases, their spread and persistence among farm animals, humans and the environment (including wildlife and plant species) [21].

The survey performed revealed a good level of biosecurity (≥50) in the majority of farms (68.4%). The average total biosecurity score recorded in our study area was moderate. This result could be explained by the fact that broiler farmers of the studied area are increasingly interested in biosecurity following the various training received. Feed companies usually organize workshops in the area to train farmers on biosecurity. This level of biosecurity is higher than what Adel et al. [36] recorded in Egypt, where 25% of farms had a good level of biosecurity. A lack of training and awareness about biosecurity and the cost needed to implement biosecurity measures can justify this difference. The biosecurity score found is higher than 38 obtained in Egypt [37] and 33 in Nepal [38]. This divergence of results could be attributed to the fact that the percentage of farmers who had received training in poultry farming was significantly higher in our study. Similar results to ours were also recorded in Sudan [39] and Bangladesh [40]. The least implemented biosecurity measures were related to feed and water [30]. This low score is due to the fact that feed silos and feed storage rooms were not completely impermeable to water, birds and pests. In addition, feed suppliers most often had direct contact with the building where the broilers were kept, and bacteriological analyses of water were not carried out in almost all the farms. Waktole et al. [4] and Elhassan et al. [39] recorded scores of 17 and 57 for biosecurity measures related to feed and water in chicken farms in Ethiopia and Sudan, respectively. This divergence highlights the diversity of chicken farming practices in Africa. Failure to comply with biosecurity measures associated with feed and water promotes the emergence and spread of pathogens such as Salmonella, which constitute a major constraint in poultry farming, reducing production performance and thus leading to significant economic losses [41]. Notably, feed and water are among the primary sources and vectors of Salmonella, facilitating its introduction and spread within the poultry production chain [41].

The results obtained showed that a high proportion of antibiotics were used by farmers for preventive purposes. In addition, wrong doses were administered for 84% of the AS used. This result could be explained by the poor knowledge of farmers about antibiotics. Insufficient knowledge of poultry farmers concerning AMU has been reported in other regions of Cameroon [9,42]. This high misuse of antibiotics will certainly result in a continuously increasing AMR in farms. Our results corroborate those of Azabo et al. [43] obtained in Tanzania, where antibiotics were used preventively by more than half of the farmers surveyed. The preventive use of antibiotics exerts a selection pressure on bacteria, favoring the emergence and spread of resistant strains of pathogens. These resistant bacteria can then be transmitted to humans through the consumption of contaminated food, contact with animals, or via the environment [44]. Critically important antibiotics were the most widely used antimicrobials in the study area. This could be explained by the fact that the majority of farmers do not have adequate knowledge about the classifications of antibiotics and the human health risks associated with their overuse. AMR to these antibiotics found in Cameroon is in line with these results [9,45]. Similar results were found in other lower–middle income economies [46,47,48]. This highlights the need to sensitize livestock farmers on the importance of the choice of antibiotics during treatments in the context where veterinarians are not always available to help farmers. The use or misuse of important human antibiotics in poultry could make infections more difficult to treat and limit the effectiveness of antibiotics in human medicine [49]. Tetracyclines were by far the most used class of antibiotics, accounting for over 30%. They are widely used in poultry farming because they have a broad spectrum of action [50], are inexpensive, and are frequently used as growth promoters in poultry farming [51]. Our results corroborate those of Ayebare et al. [52] in Uganda, where this class of antibiotics was also the most used in semi-intensive broiler farms. Bamidele et al. [46] found similar results in smallholder poultry production in Nigeria. The amount of antibiotics used to produce one kilogram of broiler meat was 784.4 mg/kg. This high amount could be due to the fact that the majority of farmers administered antibiotics for preventive purposes without conforming to the prescribed dose. In addition, they do not perform the treatment under the supervision of a qualified veterinarian. This quantity of antibiotic is much higher than those reported by Rahmatallah et al. [31] in Morocco (63.48 mg/kg), Umair et al. [53] in Pakistan (154.58 mg/kg) and Moffo et al. [27] in the West and Center regions of Cameroon (143.8 mg/kg). This difference could be attributed to the higher accessibility of veterinary drugs in the Adamawa and North Regions of Cameroon, which border Nigeria, the main source of veterinary medications for French-speaking African countries [54]. The lack of strict regulation regarding veterinary drug distribution and usage in Cameroon makes these borders porous.

Despite the fact that the quantity of antibiotics used to produce one kg of meat was relatively lower in farms with good biosecurity compared to farms with poor biosecurity, statistical analyses did not show a correlation between the level of biosecurity and the amount of antibiotics used in farms. Similarly, the amount of antibiotics used in farms with a good level of biosecurity was statistically similar to that of farms with poor biosecurity. This is certainly due to the high proportion of antibiotics used for preventive purposes. Extensive use of antibiotics can lead to the development of resistance, making these drugs less effective in treating infections [55,56]. By strengthening biosecurity practices, livestock producers can not only reduce their reliance on antibiotics but also contribute to the fight against antimicrobial resistance, which is a major public health issue. This result highlights once again the need to train farmers on antimicrobial usage. Adebowale et al. [57] and Ibrahim et al. [40] recorded a negative correlation between the level of biosecurity and the amount of antibiotics used in Nigeria and Bangladesh, respectively. This discrepancy would be due to the fact that the majority of farms surveyed in their studies used antibiotics mainly for curative purposes.

The findings showed that there was a significant but weak positive correlation (R = 0.276) between ADG and biosecurity. Indeed, good biosecurity results in healthy animals, which will allow better feed intake and digestion, thus improving weight gain. This can justify the fact that an improvement in FCR with the level of biosecurity was recorded. The logical consequence is also the positive correlation noted between the performance index and biosecurity level. These results are in agreement with the work of Ramukhithi et al. [58] in South Africa and Gelaude et al. [24] in Belgium, who also established that the application of biosecurity measures is associated with an improvement in broiler production performance. It is therefore important to raise awareness among farmers about the adoption of farm biosecurity because of its dual effects of disease prevention and improvement of production performance. Similarly, a strong negative correlation (*p* < 0.001, R = −0.689) was recorded between biosecurity and the mortality rate in broiler farms. Indeed, implementation of biosecurity measures prevents the entry or spread of pathogens on the farm, thus reducing the incidence of diseases and, therefore, reducing animal mortality [23]. This finding aligns with that of Khan et al. [59] obtained during the study conducted in the Regions of Bogura, Rangpur, and Dinajpur in Bangladesh.

The main limitation of the present study is the fact, even if though considerable effort was made to ensure accuracy, data concerning AMU were mainly collected by the farmers after the objective of the study were explained to them. This may have increased the likelihood of reporting bias.

## 5. Conclusions

This study aimed to assess the impact of biosecurity on production performance and AMU in broiler farms in Cameroon. It appears that biosecurity in poultry farms contributes significantly to improving production performance, particularly by reducing the mortality rate and improving ADG, FCR, and performance index. However, it does not significantly influence the amount of antibiotics used, due to the mainly preventive use of antimicrobials. Most of the antibiotics used were classified as critically important for human medicine and were underdosed/overdosed. This finding highlights the need to increase awareness among farmers of the importance of biosecurity and the responsible use of antimicrobials. The development and enforcement of more stringent policies to reduce the use of critically important antibiotics in livestock farms is recommended. All of these measures will help to improve the profitability of broiler farms, reduce the use of antibiotics, and minimize the risks of AMR, a critical public health concern.

## Figures and Tables

**Table 1:** Demographics of the respondents involve in the study (*n* = 57).

Variables	Number (*n*)	Percentage (%)
Gender		
Male	45	78.9
Female	12	21.1
Age (years)		
20–30	13	22.9
31–40	34	60.1
41–50	6	9.9
51–60	4	7.1
Educational level		
No education	5	9.0
Primary	8	14.5
Secondary	26	44.7
Higher	18	31.8
Training in poultry farming		
Yes	26	45.4
No	31	54.6
Years of experience in poultry farming		
2–10	47	82.6
11–20	7	11.9
21–30	3	5.5
Poultry farming as main activity		
Yes	13	24.1
No	44	75.9

**Table 2:** Biosecurity score in broiler farms (*n* = 57) in the Adamawa and North Regions (Cameroon).

Variables	Average	SD	Min	Median	Max
**External biosecurity**	**50**	**12**	**23**	**53**	**72**
Purchase of one-day chicks	48	23	9	51	100
Depopulation of broilers	38	18	17	34	72
Feed and water	30	19	5	32	77
Removal of manure and carcasses	35	25	0	45	78
Visitors and farmworkers	55	17	19	60	80
Material supply	74	29	0	100	100
Infrastructure and biological vectors	63	16	28	65	90
Location of the farm	54	20	19	61	100
**Internal biosecurity**	**56**	**9**	**31**	**59**	**73**
Disease management	66	17	23	70	100
Cleaning and disinfection	37	910	19	37	67
Materials and measurements between compartments	78	22	0	82	100
**Overall biosecurity score**	**52**	**10**	**28**	**52**	**71**

SD: standard deviation; Min: Minimum; Max: maximum.

**Table 3:** Treatment incidence of the antibiotics used in farms in Adamawa and North (Cameroon).

Variables	Active Substance	IT_UDD_ (Min–Max)	TI_ADD_ (Min–Max)
HPCIA			
Quinolones	Enrofloxacin	78.3 (66.7–111.1)	23.40 (0.1–98.8)
	Norfloxacin	90.9 (66.7–111.1)	57.7 (13.8–137.2)
Macrolides	Erythromycin	88.9 (66.7–133.3)	3.51 (1.4–5.6)
	Thiacynate		
Polymyxins	Colistin	82.4 (66.7–111.1)	51.6 (0.3–920.9)
HIA			
Beta–lactams	Amoxicillin	66.7 (66.7)	22.5 (14.4–31.0)
Tetracyclines	Oxytetracycline	87.9 (44.4–155.6)	34.4 (0.1–233.3)
	Doxycycline	91.4 (66.7–111.1)	178.8 (19.0–397.0)
Sulfonamides	Sulfadimethoxine	88.9 (66.7–111.1)	272.8 (90.0–698.1)
	Sulfadiazine	88.9 (66.7–111.1)	13.9 (6.1–25.8)
	Trimethoprim	86.5 (45.3–111.1)	18.8 (1.2–93.1)
Amphenicols	Florfenicol	44.4 (44.4)	16.3 (16.3)
CIA			
Aminoglycosides	Streptomycin	100.0 (66.7–133.3)	4.6 (3.5–5.6)
	Neomycin	90.9 (66.7–155.6)	14.2 (0.4–129.8)
Macrolides	Tylosin	90.4 (66.7–111.1)	61.8 (5.9–198.5)

The values represent the average of incidence, more or less the standard deviation. TI_ADD_: Treatment incidence with animal daily dose; TI_UDD_: Treatment incidence with used daily dose. HPCIA: highest priority critically important antimicrobials; CIA: critically important antimicrobials; HIA: highly important antimicrobials.

**Table 4:** Quantity of antibiotics used (mg) per kg of broiler meat produced in the study regions.

Variables	Active Substance	Quantity (mg)	Percentage (%)
HPCIA		113.8	14.5
Quinolones	Enrofloxacin	7.9	1.0
	Norfloxacin	27.7	3.5
Macrolides	Erythromycin thiacynate	6.8	0.9
Polymyxins	Colistin	71.4	9.1
HIA		509.5	64.9
Beta-lactams	Amoxicillin	105.0	13.4
Tetracyclines	Oxytetracycline	36.8	4.7
	Doxycycline	198.0	25.2
Sulfonamides	Sulfadimethoxine	49.1	6.3
	Sulfadiazine	98.6	12.6
	Trimethoprim	14.7	1.9
Amphenicols	Florfenicol	7.3	0.9
CIA		161.1	20.5
Aminoglycosides	Streptomycin	2.0	0.3
	Neomycin	61.4	7.8
Macrolides	Tylosin	97.7	12.5

HPCIA: highest priority critically important antimicrobials; CIA: critically important antimicrobials; HIA: highly important antimicrobials.

**Table 5:** Correctness of dosage (ratio UDD/ADD) of antibiotics used in broiler farms.

Active Substance	Underdosing (%)	Correctly Dosing (%)	Overdosing (%)	Total Number of Treatments
Amoxicilline	5 (100)	0	0	5
Colistine	43 (69.3)	6 (9.7)	13 (21.0)	62
Doxycycline	2 (22.2)	0	7 (77.8)	9
Enrofloxacine	22 (95.7)	1 (4.3)	0	23
Erythromycin thiacynate	3 (100)	0	0	3
Florfénicol	1 (100)	0	0	1
Neomycine	10 (80.9)	0	1 (9.1)	11
Norfloxacine	9 (81.8)	0	2 (18.2)	11
Oxytetracycline	56 (82.4)	6 (8.8)	6 (8.8)	68
Streptomycine	2 (100)	0	0	2
Sulfadiazine	6 (100)	0	0	6
Sulfadiméthoxine	0	0	4 (100)	4
Trimethoprime	9 (90.0)	1 (1.0)	0	10
Tylosine	11 (73.3)	0	4 (26.7)	15
Total	179 (77.8)	14 (6.1)	37 (16.1)	230

ADD: animal daily dose; UDD: used daily dose; underdosing: UDD/ADD < 0.8; correct dosing: 0.8 ≤ UDD/ADD ≤ 1.2; overdosing: UDD/ADD > 1.2.

**Table 6:** Quantities of antibiotics used based on biosecurity level.

Variable	Good Biosecurity	Poor Biosecurity
TI_UDD_	68.41 ± 38.73 ^a^	75.23 ± 40.13 ^a^
TI_ADD_	40.89 ± 24.74 ^a^	39.58 ± 25.82 ^a^
Q_kg_	35.66 ± 15.10 ^a^	47.75 ± 26.20 ^a^

For the same variable (same line), values with same letters of the alphabet (a) are statistically similar at *p* > 0.05; Q_kg_ = Quantity of antibiotics (mg) used to produce 1 kg of meat; TI_ADD_: Treatment incidence with animal daily dose; TI_UDD_: Treatment incidence with used daily dose.

**Table 7:** Correlation between AMU and biosecurity level.

Variable	R^2^	*p*-Value
TI_UDD_	−0.099	0.100
TI_ADD_	−0.180	0.771
Q_kg_	−0.008	0.889

R^2^ represents the Pearson correlation coefficient; TI_ADD_: Treatment incidence with animal daily dose; TI_UDD_: Treatment incidence with used daily dose; Q_kg_ = quantity of antibiotics (mg) used for 1 kg of meat.

**Table 8:** Production performances of the broiler farms surveyed (*n* = 57).

Variables	Average ± Standard Deviation
Average daily gain (g)	
Starter	25.35 ± 3.51 ^b^
Grower	59.56 ±13.45 ^a^
Finisher	53.34 ±13.12 ^a^
**Average**	**45.71 ± 5.14**
Feed conversion ratio	
Starter	2.39 ± 0.82 ^a^
Grower	1.69 ± 0.87 ^b^
Finisher	1.60 ± 0.51 ^b^
**Average**	**1.59 ± 0.48**
Mortality rate (%)	
Starter	1.77 ± 1.28 ^a^
Grower	1.13 ± 0.87 ^ab^
Finisher	0.76 ± 0.52 ^b^
**Total**	**3.55 ± 3.30**
**Performance index**	**317.76 ± 115.56**

For the same variable, values with different letters of the alphabet (a, b) are significantly different at *p* > 0.05.

**Table 9:** Production performances according to biosecurity level of broiler farms.

Production Performances	Good Biosecurity	Poor Biosecurity	*p*-Value
Average daily gain (g)			
Startup	26.22 ± 3.20 ^a^	25.30 ± 4.49 ^a^	0.200
Growth	62,08 ± 11,40 ^a^	54.03 ± 15.03 ^a^	0.103
Finishing	53,07 ± 14.71 ^a^	53.77 ± 11.34 ^a^	0.517
**Average**	**46.54 ± 5.18 ^a^**	**43.80 ± 4.16 ^b^**	**0.034**
Feed conversion ratio			
Startup	2.13 ± 0.39 ^a^	2.57 ± 1.08 ^b^	0.007
Growth	1.49 ± 0.52 ^a^	1.99 ± 1.16 ^a^	0.096
Finishing	1.59 ± 0.61 ^a^	1.75 ± 0.58 ^b^	0.029
**Average**	**1.50 ± 0.35 ^a^**	**1.72 ± 0.57 ^b^**	**0.026**
Mortality rate (%)			
Startup	1.10 ± 0.92 ^a^	3.16 ± 2.22 ^b^	<0.001
Growth	0.47 ± 0.16 ^a^	2.22 ± 1.85 ^b^	<0.001
Finishing	0.41 ± 0.19 ^a^	2.09 ± 1.49 ^b^	0.008
**Total**	**2.47 ± 1.42 ^a^**	**6.65 ± 3.12 ^b^**	**<0.001**
**Performance Index**	**339.21 ± 105.79 ^a^**	**268.22 ± 101.09 ^b^**	**0.015**

Farm with good level of biosecurity (total score ≥ 50%). Farm with poor level of biosecurity (total score ≤ 49%). Values in the same line with different letters of the alphabet (a, b) are significantly different at *p* > 0.05.

## Data Availability

The raw data supporting the conclusions of this article will be made available by the authors on request.

## Abbreviations

The following abbreviations are used in this manuscript:

ADG	Average daily gain
FCR	Feed conversion ratio
PI	Performance index
AMU	Antimicrobial usage
AMR	Antimicrobial resistance
TI	Treatment incidence
UDD	Daily dose used
ADD	Animal daily dose
AS	Active substance
HPCIA	Highest priority critically important antimicrobials
CIA	Critically important antimicrobials
HIA	Highly important antimicrobials
